# Developing a community facilitator‐led participatory learning and action women's group intervention to improve infant feeding, care and dental hygiene practices in South Asian infants: NEON programme

**DOI:** 10.1111/hex.13557

**Published:** 2022-07-27

**Authors:** Logan Manikam, Shereen Allaham, Isabel‐Catherine Demel, Ummi Aisha Bello, Maryan Naman, Michelle Heys, Neha Batura, Clare Llewellyn, Andrew Hayward, Rajalakshmi Lakshman, Jenny Gilmour, Kelley Webb Martin, Carol Irish, Chanel Edwards, Mfon Archibong, Corinne Clarkson, Mary Marsh, Daley Delceta, Amanda Nutkins, Lily Islam, Seema Bajwa, Sabiha Malek, Jasvir Bhachu, Geromini Pushpakanthan, Monica Lakhanpaul

**Affiliations:** ^1^ Department of Epidemiology and Public Health University College London Institute of Epidemiology and Health Care London UK; ^2^ Aceso Global Health Consultants Limited London UK; ^3^ Department of Life Sciences and Medicine King's College London GKT School of Medical Education London UK; ^4^ Specialist Children and Young People's Services East London NHS Foundation Trust London UK; ^5^ Population, Policy and Practice UCL Great Ormond Street Institute of Child Health London UK; ^6^ Institute for Global Health University College London London UK; ^7^ Department of Behavioural Sciences and Health University College London London UK; ^8^ MRC Epidemiology Unit University of Cambridge Cambridge UK; ^9^ Tower Hamlets GP Care Group Mile End Hospital London UK; ^10^ Children's Health 0‐19 Service London Borough of Newham London UK; ^11^ Waltham Forest Council London UK; ^12^ Chingford Health Centre Waltham Forest London UK; ^13^ NELFT NHS Foundation Trust London UK; ^14^ Tower Hamlets Community Facilitator London UK; ^15^ Newham Community Facilitator London UK; ^16^ Whittington Health NHS Trust London UK

**Keywords:** children, community engagement, early interventions, infant, nutrition, online engagement, participatory approach

## Abstract

**Introduction:**

The Nurture Early for Optimal Nutrition (NEON) study is a multiphase project that aims to optimize feeding, care and dental hygiene practices in South Asian children <2 years in East London, United Kingdom. The multiphase project uses a participatory learning and action (PLA) approach facilitated by a multilingual community facilitator. In this paper, we elaborate on the process and results of the Intervention Development Phase in the context of the wider NEON programme.

**Methods:**

Qualitative community‐based participatory intervention codevelopment and adaptation.

**Setting:**

Community centres in East London and online (Zoom) meetings and workshops.

**Participants:**

In total, 32 participants registered to participate in the Intervention Development Phase. Four Intervention Development workshops were held, attended by 25, 17, 20 and 20 participants, respectively.

**Results:**

Collaboratively, a culturally sensitive NEON intervention package was developed consisting of (1) PLA group facilitator manual, (2) picture cards detailing recommended and nonrecommended feeding, care and dental hygiene practices with facilitators/barriers to uptake as well as solutions to address these, (3) healthy infant cultural recipes, (4) participatory Community Asset Maps and (5) list of resources and services supporting infant feeding, care and dental hygiene practices.

**Conclusion:**

The Intervention Development Phase of the NEON programme demonstrates the value of a collaborative approach between researchers, community facilitators and the target population when developing public health interventions. We recommend that interventions to promote infant feeding, care and dental hygiene practices should be codeveloped with communities. Recognizing and taking into account both social and cultural norms may be of particular value for infants from ethnically diverse communities to develop interventions that are both effective in and accepted by these communities.

**Patient and Public Involvement and Engagement:**

Considerable efforts were placed on Patient/Participant and Public Involvement and Engagement. Five community facilitators were identified, each of which represented one ethnic/language group: (i) Bangladeshi/Bengali and Sylheti, (ii) Pakistani/Urdu, (iii) Indian/Gujrati, (iv) Indian/Punjabi and (v) Sri Lankan/Tamil. The community facilitators were engaged in every step of the study, from the initial drafting of the protocol and study design to the Intervention Development and refinement of the NEON toolkit, as well as the publication and dissemination of the study findings. More specifically, their role in the Intervention Development Phase of the NEON programme was to:

1. Support the development of the study protocol, information sheets and ethics application.
2. Ensure any documents intended for community members are clear, appropriate and sensitively worded.
3. Develop strategies to troubleshoot any logistical challenges of project delivery, for example, recruitment shortfalls.
4. Contribute to the writing of academic papers, in particular reviewing and revising drafts.
5. Develop plain language summaries and assist in dissemination activities, for example, updates on relevant websites.
6. Contribute to the development of the NEON intervention toolkit and recruitment of the community members.
7. Attend and contribute to Intervention Development workshops, ensuring the participant's voices were the focus of the discussion and workshop outcomes.

## 1 INTRODUCTION

The first 1000 days of a child's life are an important period for both growth and brain development. There is mounting evidence to show that influences during pregnancy (such as high maternal prepregnancy body mass index, excess gestational weight gain, gestational diabetes, tobacco exposure) and infancy (e.g., high infant birth weight or accelerated infant weight gain) may alter lifetime risk of nutrition and dental‐related diseases.^1^ Feeding practices developed during this critical period can therefore impact a child's nutrition, growth, dental health and cognitive development. Healthy, age‐appropriate feeding practices are known to be associated with decreased risk of heart disease, obesity and diabetes in the long term, with suboptimal practices having the opposite effect.^2^

Studies in the past have shown that Britain's minority ethnic population is most disadvantaged across a range of social and economic outcomes. These social and economic inequalities underpinned by ethnicity are fundamental causes of many health inequalities amongst different ethnic groups in the United Kingdom. Some of the widest disparities have been observed to be in the South Asian population, with the Pakistani and, to an even greater extent, the Bangladeshi communities, standing out as the most disadvantaged compared to other ethnicities.^3^

The World Health Organization (WHO) Infant and Young Children Feeding Guidelines have been adopted by many countries. Despite this uptake, a systematic review of studies assessing feeding practices, and the sociocultural beliefs that underpin them, in children <2 years old within South Asian families living in the United Kingdom, India, Pakistan and Bangladesh show that nonrecommended complementary feeding practices continue to be followed.^4^, ^5^, ^6^, ^7^ Further, adult migrants from low‐ and middle‐income countries (LMICs) moving to high‐income countries (HICs) have been shown to be at increased risk of developing metabolic disorders, such as obesity and type II diabetes compared to the host population.^8^ Factors that affect these feeding practices persisting following migration included bicultural issues or low acculturation levels and conflicting information provided by health professionals and the information shared by extended family, and religious and community leaders in which the latter have an influencing role in affecting feeding practices within the South Asian Community.^4^, ^5^, ^6^ In contrast, barriers to engagement with WHO‐recommended complementary feeding practices in South Asian countries included conflicts about the best use of mothers' time, short birth intervals and poverty.^4^, ^5^, ^6^

Based on the findings that highlight the need for targeted early‐life interventions, and its potential to prevent the development of short‐ and long‐term outcomes and reduce lifetime health inequalities. Further, evidence about the drivers of non‐recommended feeding practices among recently migrated South Asian communities in HICs suggests that tailored, culturally sensitive interventions have the potential to change behaviours, thereby reducing children's increased risk of developing metabolic disorders later in life.^7^

At present, however, the number of such tailored and culturally sensitive interventions is limited, most are developed for the majority population.^9^ Health services in the United Kingdom continue to provide mostly unidirectional information based on guidelines and NHS recommendations to parents/carers on the optimal nutrition, care and dental hygiene practices for their children. Specific ethnic groups may be thus marginalized by this universal approach as most of the advice is not tailored to their cultural practices. This inevitably results in an inability to appropriately support families in skills development, which is key to providing optimal infant feeding, care and dental hygiene support. Respecting the seven theoretical domains framework of intervention acceptability (i.e., affective attitude, burden, perceived effectiveness, ethicality, intervention coherence, opportunity costs and self‐efficacy) would further support the design of a tailored approach that optimizes intervention acceptability and hence, uptake.^10^ Considering the limited NHS resources and the NHS 10‐year Forward Plan, which aims to shift the emphasis to the community, there is a clear need for low‐cost culturally sensitive interventions that utilize a strengths‐based approach.

Community‐based participatory research may be one effective way to design and implement culturally targeted interventions successfully.^11^ The Participatory Learning and Action (PLA) group approach, one form of participatory research, is a low‐cost, community‐based, intervention mostly employed in LMICs.^12^, ^13^, ^14^, ^15^ It involves an iterative process whereby multilingual facilitators guide participants through a four‐stage cycle of identifying and prioritizing contextual issues, designing strategies to address these issues, implementing these strategies and a postimplementation evaluation.^16^ It views research as a collective enterprise between researchers and the researched, taking place in familiar community settings.^11^ By empowering the target population and making use of local community assets where possible, the PLA approach has the potential to be applied and adapted to different population groups and topic areas. Evidence from a number of studies has shown positive effects of using a PLA approach. In a meta‐analysis of seven cluster randomized controlled trials (RCTs) in LMICs, PLA was shown to be effective, reducing maternal and neonatal mortality by 37% and 23%, respectively.^12^ A RCT has also demonstrated PLA groups to reduce maternal depression, increase exclusive breastfeeding rates, and decrease under five morbidity.^14^ Additionally, in a Mumbai‐based RCT, PLA groups achieved improvements in maternal practices and care‐seeking behaviour.^15^

This strategy's success in an LMIC setting led to its official endorsement by WHO as a cost‐effective strategy to improve maternal and infant survival in rural low‐resource settings.^17^ Considerable evidence has since been published on the feasibility of adapting reverse innovations for implementation in other resource‐constrained settings;^18^, ^19^ however, the applicability and feasibility of this approach in an HIC context, such as that of the United Kingdom, remains relatively unexplored.

The Nurture Early for Optimal Nutrition (NEON) programme was created with the aim to improve infant feeding, care and dental hygiene practices in South Asian minority groups using a community‐based, participatory approach.

The NEON programme consists of two stages. The first stage entailed a formative and feasibility study, which focused on the Bangladeshi population in Tower Hamlets in East London.

The findings from the first stage of the programme, led to the continuation of the programme, and the refinement of associated interventions, in a second stage of the NEON programme comprising of Intervention Development. In the second stage in addition to Tower Hamlets in East London, we expanded to two additional boroughs, namely Newham and Waltham Forest.

This paper focuses on the Intervention Development Phase of the second stage of the NEON programme, involving the participatory approach for the codevelopment of the NEON Intervention toolkit with South Asian community members to optimize infant feeding, care and dental hygiene practices within South Asian infants aged <2 years in East London.

The feasibility of delivery of the codeveloped NEON intervention toolkit, and the geographical scalability of the NEON programme more generally, will be tested in the following phase of the NEON Pilot Feasibility Randomized Controlled Trial.

## 2 MATERIALS AND METHODS

### 2.1 Setting and target population

The initial development of the NEON programme was conducted in the London Boroughs of Tower Hamlets between 2015 and 2018.^20^ Following a sequential design for evidence generation, the first stage of the NEON programme involved an initial systematic literature review on infant feeding and care practices in Bangladeshi and other South Asian families living in India, Pakistan, Bangladesh and high‐income setting,^4^, ^5^, ^6^, ^7^ followed by a qualitative formative study phase.^20^ Evidence from both the literature and the subsequent qualitative study informed the design and adaptation of the PLA approach to optimize infant feeding and care practices among Bangladeshi infants aged 6–23 months. A subsequent wider development was carried out between December 2019 and March 2021, in the study boroughs in Tower Hamlets, Newham and Waltham Forest in East London. Each aforementioned borough comprises some of the largest and fastest‐growing, yet equally most socially and economically deprived, ethnic populations in the United Kingdom.^21^

Given the relatively limited percentage of fluent English speakers in the selected study boroughs and NEON's dedication to design culturally sensitive interventions, one of the key drivers for participation represented shared language and culture. To enable the community engagement activities and community mobilization, participants in all study phases of the NEON will be recruited into Intervention Development meetings and workshops based on ethnicity and language. While South Asians in Tower Hamlets, Newham and Waltham Forest are known to communicate in several hundred languages,^22^, ^23^, ^24^ we used the office for national statistics (ONS) data and discussions with the local stakeholders and community facilitators to identify the most prevalent languages. These NEON ethnic/language groups are: (1) Indian/Gujarati, (2) Indian/Punjabi, (3) Pakistani/Urdu, (4) Bangladeshi/Bengali‐Sylheti and (5) Sri Lankan/Tamil.

### 2.2 Community facilitators

Community facilitators are female local facilitators with experience in running community groups who are leaders in their community. When trying to identify suitable community facilitator candidates, the following inclusion criteria were considered:

1. Females who have at least one child (preferably <24 months),
2. from the South Asian community living in the East London boroughs,
3. able to read, write and speak fluently in English and one of the other commonly spoken languages (Gujarati, Punjabi, Bengali, Sylheti, Urdu, or Tamil),
4. understand social norms and values of South Asian culture,
5. known to and respected by their local community,
6. motivated to address issues related to infant growth and development and
7. able to manage a group and have some leadership qualities.

With the help of local stakeholders and our links within the community established during the previous stage of the NEON programme (NEON formative and feasibility study with the British Bangladeshi community in Tower Hamlets to optimize infant feeding and care practices), five suitable community facilitators were recruited, each of which represented one of the aforementioned ethnic/language groups of the NEON target population. One‐day training was provided to the community facilitators before the start of the first Intervention Development workshops. This included teaching facilitation skills, note‐taking and taking informed consent.

### 2.3 Community members

South Asian residents across Tower Hamlets, Newham and Waltham Forest were recruited to take part in several Intervention Development workshops, detailed in Table 1 below.

**Table 1:** Intervention Development meetings—participants and objectives

Intervention Development meeting number	Objectives
Meeting 1	Induction and overview of the NEON study.
Meeting 2	Community map. To review the Community Asset Map adding local assets recommended by community members to providing services and support to aid infant feeding, care and dental hygiene.
Meeting 3	Conceptual map.
Meeting 4	Providing cultural infant recipes.
Meeting 5	To review the picture cards providing information on recommended and nonrecommended feeding, care and dental hygiene practices and barriers and solutions for infants <2 years old.
Meeting 6	To review the picture cards providing information on recommended and nonrecommended feeding, care and dental hygiene practices and barriers and solutions for infants <2 years old.
Meeting 7	To review the list of resources and services supporting infant feeding, care and dental hygiene practices. Consensus on all tools.
Meeting 8	Meeting with the public health infant nutritionist to further review the recipes book.

Abbreviation: NEON, Nurture Early for Optimal Nutrition.

Inclusion criteria for community members were:

1. Mothers/carers (any age) of an infant aged <24 months,
2. resident in Tower Hamlets, Newham or Waltham Forest and
3. from the South Asian background (Indian, Pakistani or Sri Lankan in Newham and Bangladeshi in Tower Hamlets).

The community members were recruited through the community facilitators' informal networks and word‐of‐mouth, and through the online campaigns on social media platforms (e.g., Facebook, Instagram).

### 2.4 NEON programme—Intervention Development Phase design

**Figure 1:**
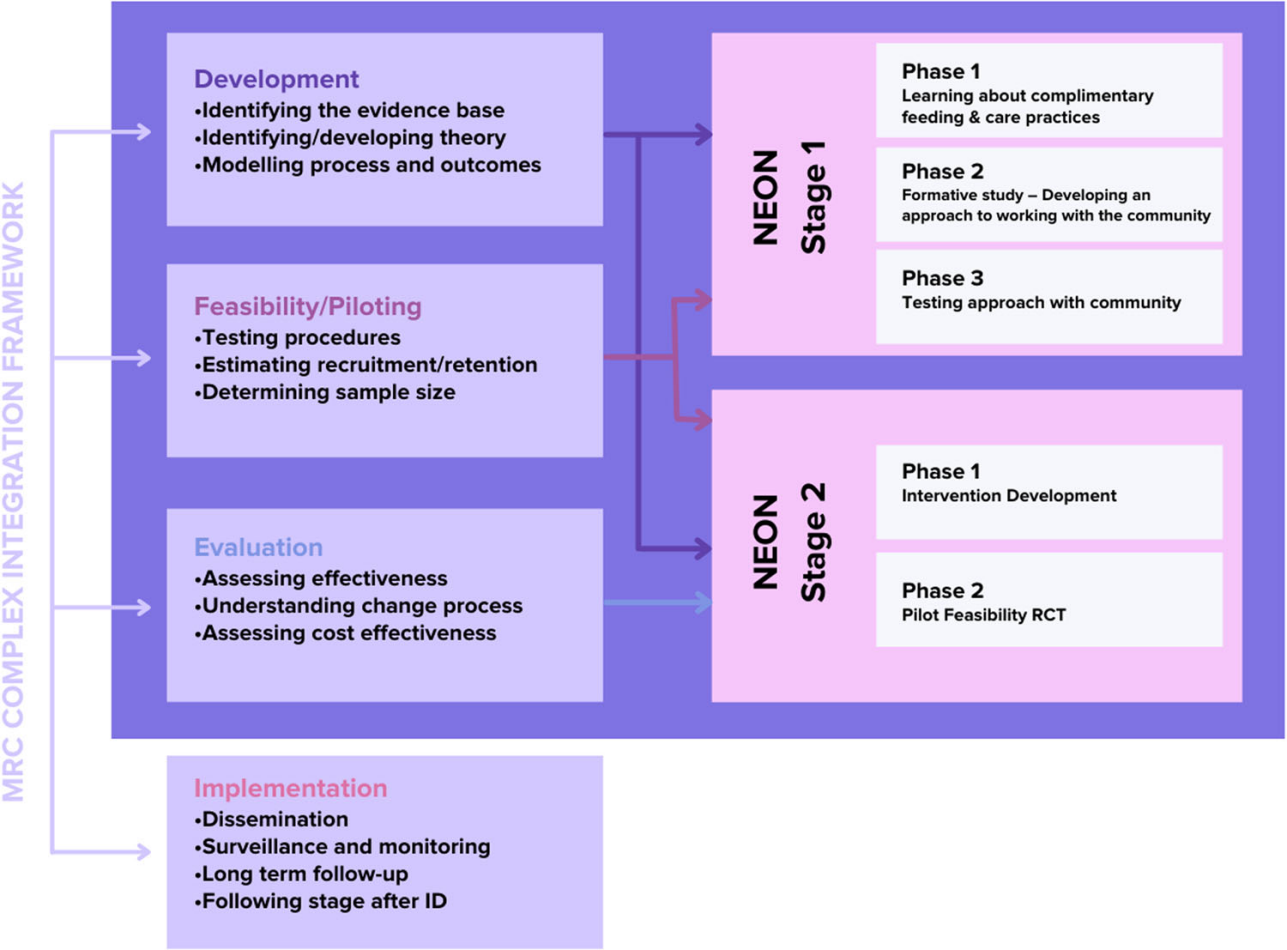
NEON programme—overview of study phases integrated Medical Research Council (MRC) complex intervention framework. NEON, Nurture Early for Optimal Nutrition.

**Figure 2:**
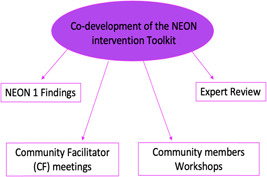
NEON—Phase 1, Intervention Development overview. NEON, Nurture Early for Optimal Nutrition.

The NEON Intervention Toolkit was designed and adjusted in a participatory approach involving four steps: (1) the integration of NEON literature review and qualitative findings, (2) Intervention Development meetings with the community facilitator, (3) Intervention Development workshops with the wider community members, followed by (4) a final review by experts and the NEON Core Team. Figure 1 illustrates the iterative process involved in the NEON Intervention Development Phase. Figure 2 shows an overview of Phase 1 of the NEON Intervention Development.

**Figure 3:**
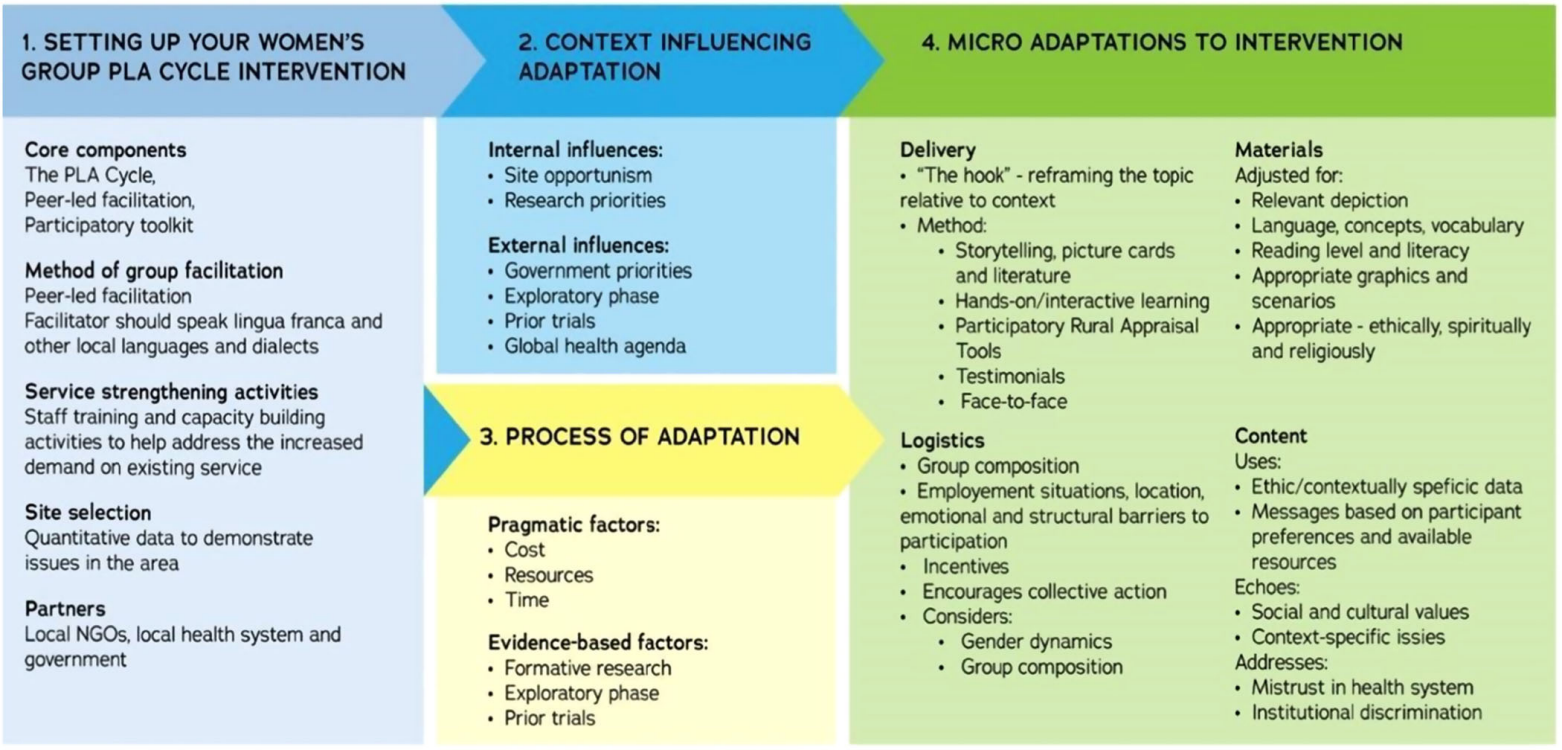
Theoretical adaptation framework for the women's group PLA cycle. PLA, Participatory Learning and Action.

Furthermore, the Theoretical Adaptation Framework^25^ for the adaptation of the Women's Group PLA cycle informed the Intervention Development toolkit and the NEON PLA group intervention. This is outlined in Figure 3 below.

### 2.5 NEON formative and feasibility study findings

In the NEON formative and feasibility study, the pilot ‘Participatory Learning’ group intervention tested three PLA group meetings. The intervention was developed through a series of workshops with community and local stakeholders. They were facilitated by consultants from Women and Children First, all of whom were experts in the design and implementation of the PLA group approach. As a result, the community facilitator NEON intervention toolkit for the three PLA group meetings was developed.^20^

Based on the qualitative data collected at the end of the NEON feasibility study,^20^ the adapted intervention was largely acceptable with positive perceptions identified amongst all participants across varying socioeconomic and demographic backgrounds. Almost all participants, including those attending one PLA group meeting, reported having introduced recommended changes into their infant feeding and care practices following the NEON intervention.

A list of recommendations and proposed solution activities was obtained from participants in the NEON feasibility study, which were subsequently translated into a modification of our suggested intervention for the subsequent Intervention Development Phase. These included:

2.5.1

*Meetings and content*—Findings from meetings and content of the NEON feasibility study recommended that participants should be given the chance to identify their own practices, barriers and solutions before viewing the picture cards. Moreover, the length of meetings should be limited to 2 h. Additionally, meeting components and contents of picture cards should be reduced and overlapping information should be removed.

2.5.2

*Improving participation*—To improve participation, NEON feasibility findings encouraged picture cards to be developed and provided in the languages of the target populations. Additionally, it was suggested to develop methods and platforms for sharing the NEON intervention package as a digital resource.

2.5.3

*Solution activities*—to enable interactive codelivery, key solution activities should be preplanned. Additionally, solution activities aimed at supporting both healthy infant food preparation and infant feeding styles should be provided. Management of the influence of extended family should be developed better. Solution activities that enable health professionals (health visitors, general practitioners (GPs), dieticians and/or dentists) to attend key sessions to provide professional advice should be provided. Finally, it was suggested to include Community Asset Maps that signpost local resources, such as healthy infant food stores (e.g., grocery stores) and play areas, in the study boroughs.

2.5.4

*Evaluation*—Measures should be identified and developed to better record the learning of groups and processes of problem‐solving. Additionally, measures to assess the social diffusion of NEON interventions through participants' friends and family networks should be identified and developed.

### 2.6 Intervention Development meetings

The Intervention Development team consisted of a PLA expert and five South Asian multilingual community facilitators from the South Asian community in East London. A total of eight Intervention Development team meetings were conducted between February 2020 and November 2020 to discuss the overview of the Intervention Development Phase and codevelop the NEON toolkit. A table summarizing the participants and objectives of each of the eight meetings is outlined in Table 1 below.

Initially, these meetings were delivered face‐to‐face in community centres. Due to COVID‐19 restrictions, the intervention was later adapted to be delivered online, utilizing Zoom software, to allow a continuation of the study despite lockdown measures.^26^ To prepare for virtual delivery of the NEON content and facilitate the NEON intervention meetings in the next feasibility RCT study, the community facilitators received group and 1:1 training from the NEON team on how to use Zoom. During these meetings, the Intervention Development team codeveloped the NEON intervention.

### 2.7 Intervention Development workshops

In preparation for the Intervention Development workshops, the five community facilitators received online training on facilitation skills, including guidance on how to obtain written or verbal consent from the community members. In the timeframe of December 2020 to February 2021, a total of four workshops took place online using the Zoom platform. Each meeting lasted for 2h, and was conducted by the community facilitators under the supervision of the NEON research fellow. The main focus of each workshop was the review and validation of one component of the intervention toolkit with wider South Asian community members of Tower Hamlets, Newham and Waltham Forest. The objectives and number of participants for each workshop are summarized in Table 2 below.

**Table 2:** Intervention Development workshops—participants and objectives

Workshop number	Objectives
Workshop 1 (*n* = 25)	To review the community assets map adding local assets recommended by community members to provide services and support to aid infant feeding, care and dental hygiene.
Workshop 2 (*n* = 17)	To review the healthy baby recipes book that provides culturally sensitive South Asian recipes and general recommendations for infants <2 years old.
Workshop 3 (*n* = 20)	To review the picture cards providing information on recommended and nonrecommended feeding, care and dental hygiene practices and barriers and solutions for infants <2 years old.
Workshop 4 (*n* = 20)	To review a list of resources and services supporting infant feeding, care and dental hygiene practices. Consensus on all tools.

The recruitment of participants was achieved through snowballing using the community facilitators network as well as online advertising campaigns using social media platforms (e.g., Facebook, Instagram). These advertisement posters were codeveloped in English and translated to the local languages of our South Asian target population (Gujarati, Punjabi, Urdu, Bengali and Tamil).

### 2.8 Experts' review

The public health infant nutritionist, public health dentist, health visitors, GPs, midwives, NEON core team and NEON steering team have been at the forefront of ensuring that the NEON toolkits are valid through thorough review and proofing. All intervention components, apart from Healthy Baby Recipes, were reviewed by experts after the Intervention Development workshops with community facilitators and community participants. Healthy Baby Recipes went through expert review after Intervention Development meetings and was validated during Intervention Development workshops by reviewing the Healthy Baby Recipes with the wider community members, followed by a final review by the experts (public health nutritionists, health visitors, midwives, academics).

### 2.9 Conceptual framework

A NEON conceptual map was first codeveloped based on the findings from the previous stage of the NEON programme (the NEON formative study)^16^ and later refined through (1) the input from NEON South Asian community facilitators and (2) the review of the NEON Core Team consisting of experts in the fields of infant nutrition, care and dental hygiene practices. Each colour on the map represents a level of the socioecological model through first, reviewing the literature, second, using findings from the NEON formative and feasibility study, third, input and comments from our NEON South Asian community facilitators and finally, refining the conceptual map with the NEON core team and incorporating their feedback and input. The conceptual map illustration is shown in Supporting Information: File S1.

Based on the Dahlgren‐Whitehead socioecological model, this map highlights the individual, sociocultural and environmental factors that were found to influence infant feeding, care and dental hygiene practices in our target population (e.g., South Asian origin infants aged <2 years in East London).

### 2.10 Data collection

Data collected on recruitment of community members included the total number of participants attending the workshops, number of community facilitators participating, participant retention and losses to follow‐up. Audio/video recordings of the Intervention Development workshops were collected using Zoom. Participant demographic questionnaires were utilized to collect demographic and socioeconomic data from eligible candidates who have agreed to take part in the study. These include gender, ethnicity, number of children, employment status, income, age and level of education. The participants were asked for their preferred language to communicate during the workshops, and all the participants preferred to communicate in English with the support of the community facilitators to explain to any participants if anything was not clear in the local language.

Data on the NEON toolkit was collected during Intervention Development meetings with Community Facilitators and experts, as well as during Intervention Development workshops with community participants and community facilitators. These workshops were recorded to aid in the extraction of recommendations and suggested changes to be incorporated in the final development of the NEON intervention toolkit.

### 2.11 Data analysis

A nominal group technique (NGT) was employed to achieve a group agreement on all the toolkits and contents developed during this phase. The NGT is a structured method which facilitates an effective agreement on the intervention toolkit and content.^27^ This was discussed with all members and voting on each component took place. Each ethnic/language group ranked each component on a scale of 1–5 with 1 being least representative and 5 being most representative for the group.

## 3 RESULTS

In total, 32 participants signed up to take part in the Intervention Development workshops. A total of 25 participants in total took part in the first Intervention Development workshop, 17 took part in the second Intervention Development workshop, and 20 took part in the third and the fourth Intervention Development workshop. Table 3 shows the characteristics of the study participants.

**Table 3:** Community members (participants) characteristics

Characteristics	Participants (*n* = 32)
Ethnic/language	
Bangladeshi/Bengali and Sylheti	8
Indian/Gujarati	5
Indian/Punjabi	7
Pakistani/Urdu	9
Sri Lankan/Tamil	3
Marital status	
Married	29
Separated	1
Widowed	1
Single	1
Relationship to the infant	
Mother	24
Aunt	2
Grandmother	6
Employment	
Full‐time employed	4
Part‐time employed	6
Nonemployed	22
Household income	
£0–£5475	7
£5475–£12,097	2
£12,098–£20,753	2
£20,754–£31,494	2
£31,495 or more	5
Not disclosed	14

In a collaborative approach, the NEON core team, community facilitators and participants of our target population developed and revised a culturally sensitive NEON intervention toolkit consisting of:

1. Picture cards.
2. Healthy Baby Recipes book.
3. Community Asset Map.
4. List of local resources and services supporting infant feeding, care and dental hygiene practices.

Quality is assured by experts (e.g., dentists, health visitors, dieticians and medical practitioners). It is envisaged that (2) and (3) will become trusted community resources.^28^

### 3.1 Picture cards

The picture cards represent one of the key educational resources for PLA group facilitators. Aimed as tools to stimulate discussion and problem‐solving during group exercises with participants, they were initially designed as visual and thematic prompts displaying information related to feeding, care and dental hygiene practices.

Their initial design was based on findings from the NEON formative study and was codeveloped and used during the first stage of the NEON programme during the NEON feasibility study.

A further refinement of the picture cards was done based on the NEON feasibility study findings and as part of the Intervention Development meetings and workshops in the NEON Intervention Development Phase. The content was further revised, in an iterative codevelopment process involving the community facilitators whose input aided in the simplification of all 45 cards and the removal of five cards that provided repetitive information.

In a third and final step, the revised picture cards were reviewed by a PLA expert who suggested merging and condensing them into four subcategories, outlined below:

1. Recommended feeding, care and dental hygiene practices.
2. Nonrecommended feeding, care and dental hygiene practice.
3. Barriers to feeding, care and dental hygiene practices.
4. Solutions to feeding, care and dental hygiene practices.

During the third workshop with the wider South Asian community members, the picture cards were shared with the community to gain their perspective and improvement suggestions asking them to allow further refinement of both the content of the cards and the pictures associated with each card.

In a final step, the picture cards were subject to review by the NEON Core Team and experts in the field (dentists, nutritionists and health visitors).

The picture cards are subsequently to be utilized in the next NEON Pilot feasibility RCT phase.

### 3.2 Healthy Baby Recipes

The Healthy Baby Recipes are designed as a resource providing advice on and ideas for age‐appropriate, culturally sensitive meals for South Asian infants.

As all of the components of the NEON toolkit, the Healthy Baby Recipes were created and refined in a participatory approach between community facilitators, community members, as well as the infant public health nutritionist (H. C.), the NEON Core Team and experts in the field of Infant nutrition.

**Figure 4:**
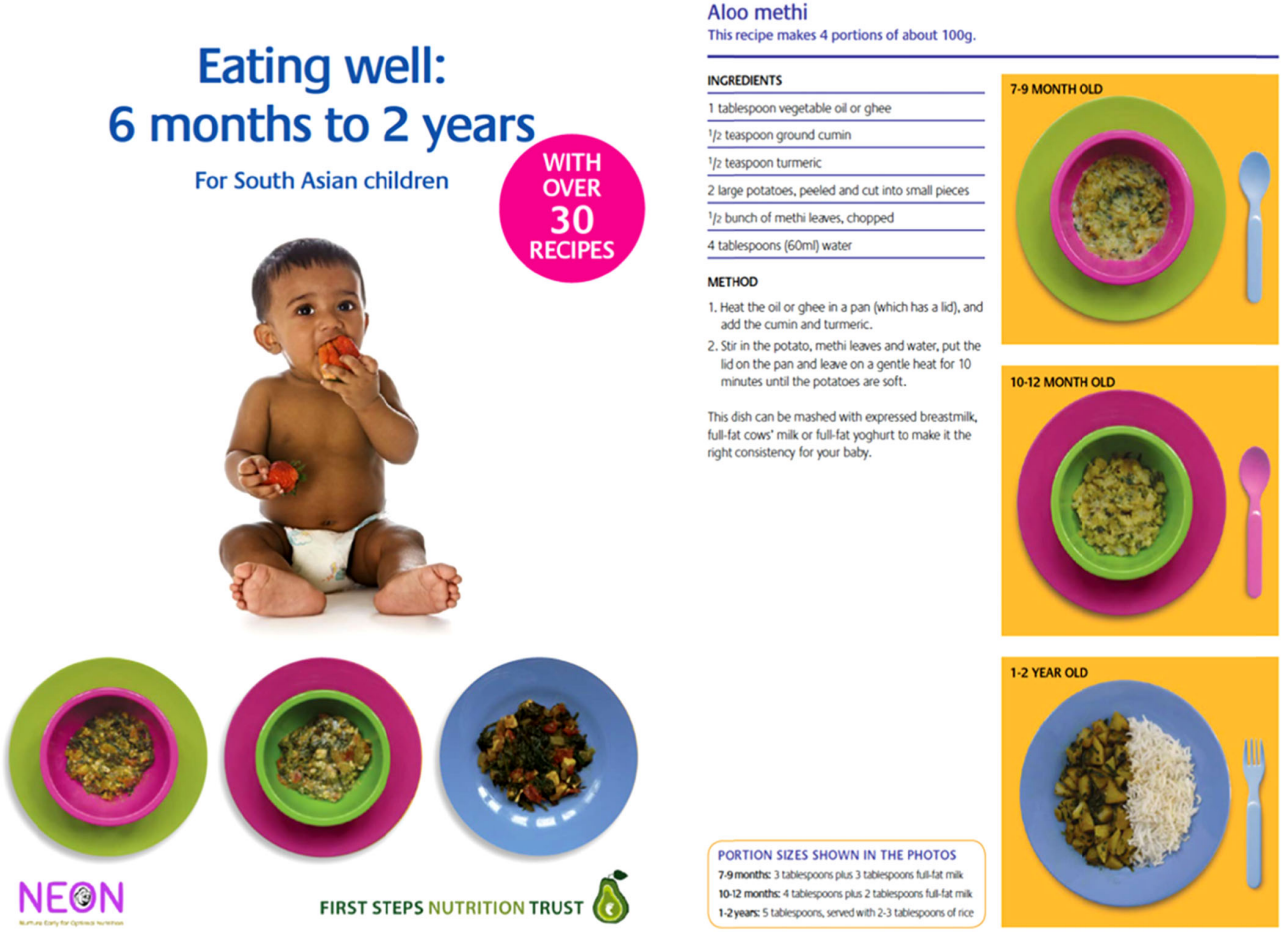
NEON tool—Healthy Baby Recipes. Sample page from healthy cultural recipes book. NEON, Nurture Early for Optimal Nutrition.

As part of the Intervention Development meetings, the selected NEON community facilitators were initially asked to provide cultural recipes for infants aged <2 years commonly used in their community. To ensure alignment with food requirements and international WHO recommendations for each infant age group (e.g., 6–9 months, 9–18 months, etc.), an infant public health nutritionist (H. C.) was consulted to review the suggested recipes and provide further suggestions. Together with an infant public health nutritionist at First Steps Nutrition, the NEON team revised the recipe contents and pictures. Furthermore, community facilitators were made aware of, and explained the rationale behind, the changes made so as to ensure this information is also passed on to the target population. In the eighth Intervention Development meeting, both recommended and nonrecommended practices were discussed in detail, highlighting the key questions that the community has about optimal nutrition for the infant. Thereafter, the community members in the second workshop reviewed the recipe book and suggested final amendments in terms of layout and presentation, as well as the title of some recipes so as to assure that they are both visually pleasing and culturally appropriate. A final revision and review of the recipe book were undertaken by the NEON core team and the steering team. Figure 4 illustrates the NEON tool for Health Baby Recipes.

### 3.3 Community Asset Map

The Community Asset Map is a tool that lists and visually displays key locations related to infant feeding, care and dental hygiene practices in East London. Local assets were suggested by community facilitators, community members and experts during one of the Intervention Development meetings. Collaboratively, places and locations thought to shape infant feeding, care and dental hygiene practices were identified. The list of providers was later subcategorized based on the type and colour coordinated as follows: GPs and healthcare centres (red), children/community centres (green), community and worship places (purple), parks (yellow) and others (brown).

The first draft of NEON's Community Asset Map tool was then refined by community members during the first Intervention Development workshop. Participants were asked to review the places on the map for each category and their suggestions for any other places that they considered to be influential in feeding, care and dental hygiene practices.

Based on the community members' feedback, the Community Asset Map was modified and later reviewed by the NEON core and steering team. Given their familiarity with the locality of the study boroughs and the community, this expert review acted as a final checking system and lead to the addition of the food banks to the Community Assets Map.

**Figure 5:**
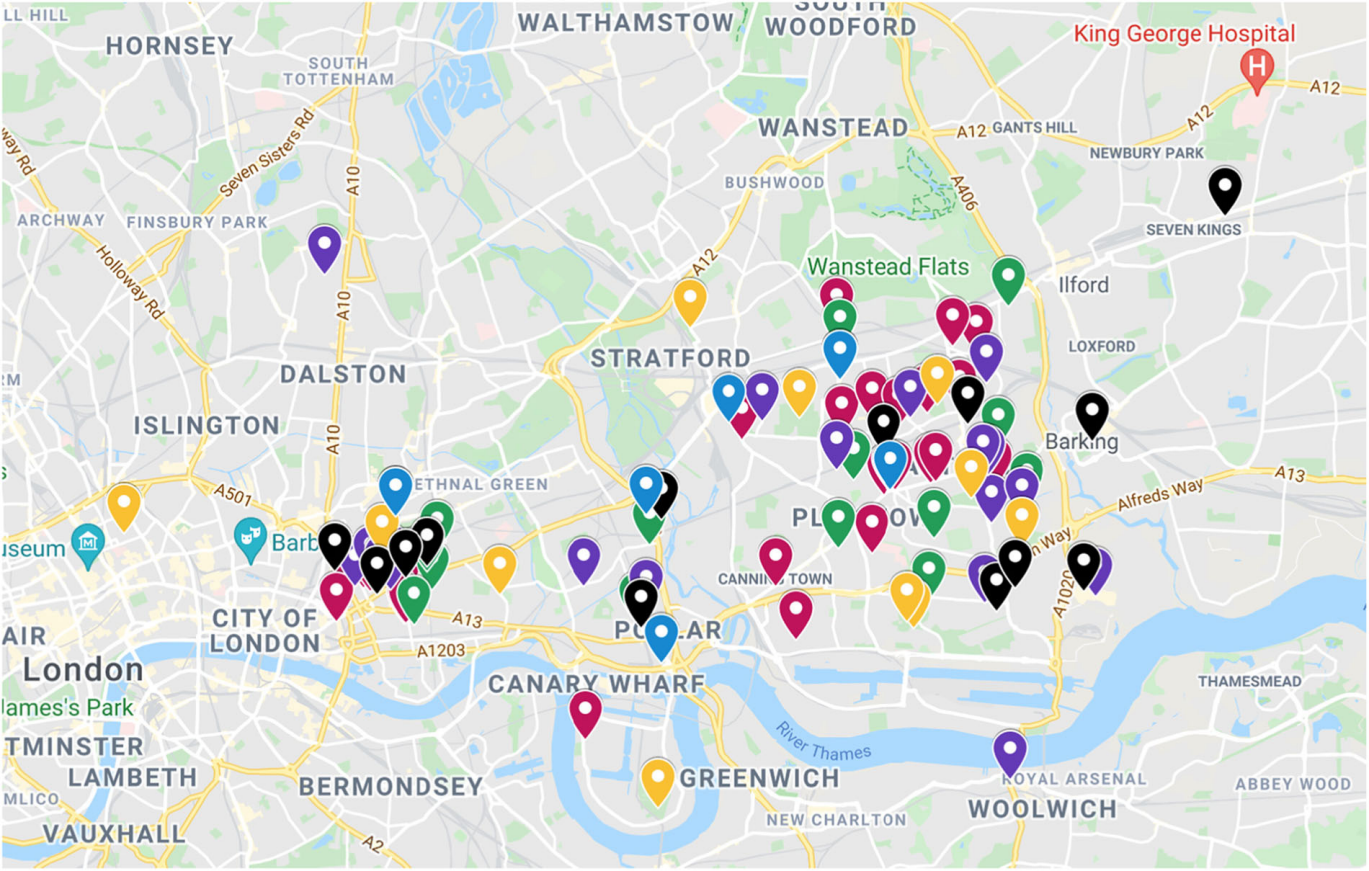
NEON Community Assets Map. NEON, Nurture Early for Optimal Nutrition.

To verify the modifications and gain further input, the community facilitators were asked to review the tool, adding key local food banks in the community (black). Figure 5 represents the final version of the NEON assets map.

### 3.4 List of local resources and services

The ‘List of local Resources and Services’ tool was initially drafted as part of the NEON feasibility study. It represents a list of available resources and services initially in Tower Hamlets supporting healthy maternal, infant and child health and development.

Codeveloped with the community facilitators during the Intervention Development meetings, this tool summarizes relevant and recommended online resources and mobile applications. The tool also entails services, such as local charities, initiatives and projects in the three target boroughs. Through the close collaboration with the community facilitators and community members, all relevant local resources and services supporting infant feeding, care and dental hygiene practices were identified and grouped into the following categories: (1) NHS and council's advice and support, (2) guidance documents and online resources and apps, (3) other local initiatives, charities and projects in East London.

**Figure 6:**
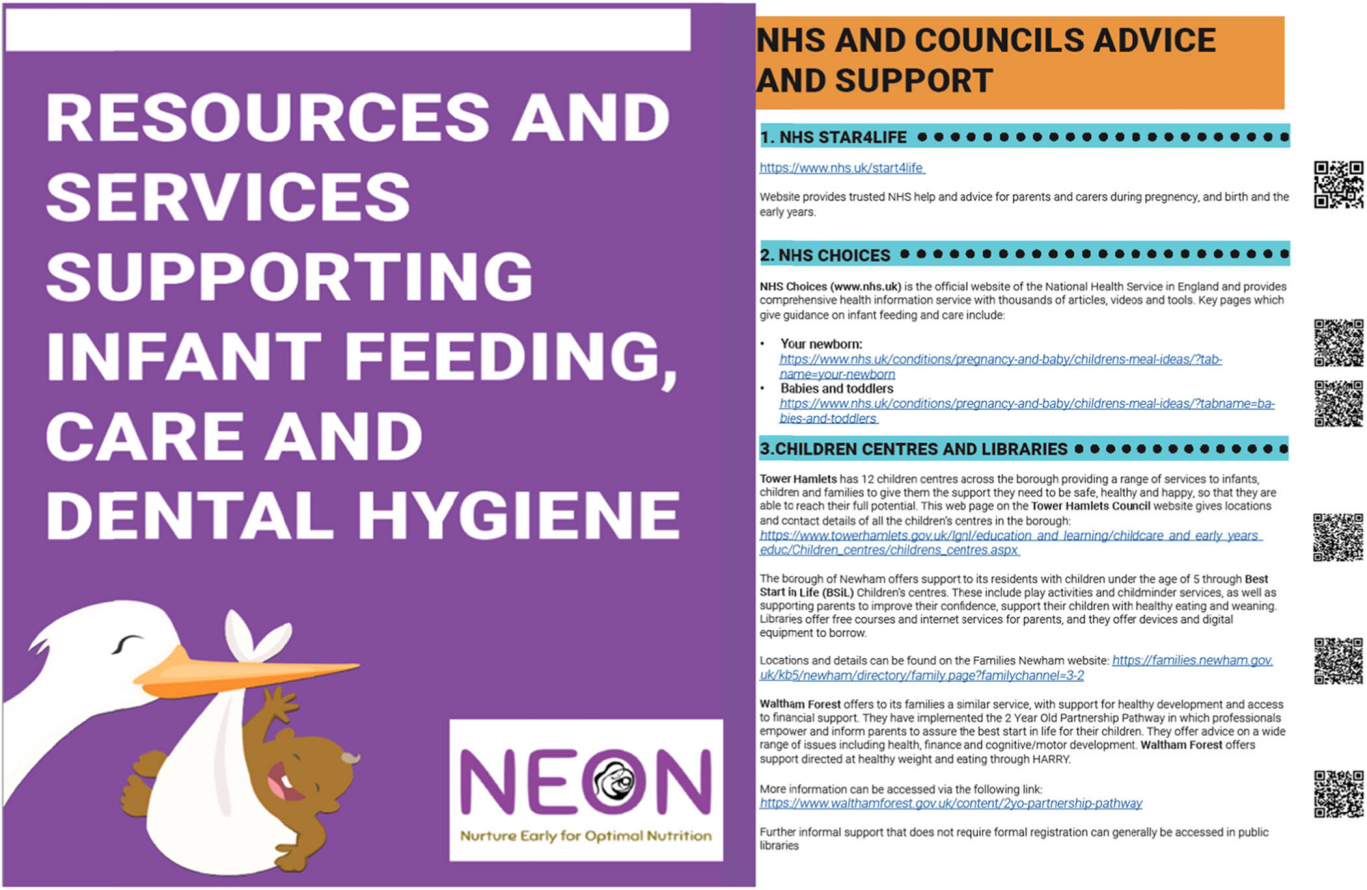
NEON list of local resources and services. NEON, Nurture Early for Optimal Nutrition.

Subsequently, this tool underwent further validation and refinement with the community members, resulting in changes in visual aspects of this tool, for example, colour and pictures, as well as the addition of a translation to specific resources that provide their services in the local language. Furthermore, a QR code to allow virtual access irrespective of time and location was incorporated following input during the fourth Intervention Development workshop. Figure 6 shows the list of local resources and services

### 3.5 Digital platform (Eredbook)

Based on the findings from the NEON feasibility study suggesting the use of a digital platform to provide access to the NEON toolkit, it was decided to make all resources available in a virtual format for the pilot feasibility RCT participants. This was achieved by collaborating with an online health and education platform, Eredbook—an NHS application‐based digital version of the paper Redbook, providing a solution for parents by enabling them to review their children's NHS records while simultaneously acting as an additional resource for health information and guidance from the NHS and other healthcare sources. In the target boroughs of Tower Hamlets, Newham and Waltham Forest, the NEON intervention toolkit will be included in the application hence allowing participants to conveniently access the available resources irrespective of location and time.

## 4 DISCUSSION

The recent WHO–UNICEF–Lancet Commission stressed the importance of placing children's health and development at the centre of the sustainable development goals (SDGs).^29^, ^30^ To achieve this, the commission proposed that alongside government investments, citizen participation and community action should be cultivated. One such approach is ‘PLA’, a strategy advocated by WHO for improving maternal, newborn and child health, particularly in rural settings in LMICs with limited access to health‐related services.^30^ This community‐led approach builds on reversals in the relation of power between community members and outsiders.^31^ Through the PLA approach, participants are encouraged to discuss their problems and needs, for example, during pregnancy and infancy, identifying potential barriers to accessing support and developing common solutions.^32^

The NEON programme highlights the wider impact that socioeconomic and cultural factors can have on early infant development—an association with a potential link to the long‐term effects seen within the target population's health outcomes, for example, the development of type 2 diabetes. Furthermore, the NEON programme has also opened the dialogue and assessed the feasibility and implementation of PLA as a mechanism to help cross‐cultural communication regarding healthcare across different communities.^32^ This is particularly important when you consider the adverse health outcomes linked to migrant populations within HIC settings.

The NEON programme aims to tackle the commission's recommendations to address SDGs 3, 10 and 11 (e.g., SDG 3: Ensure healthy lives and promote wellbeing for all at all ages; SDG 10: Reduce inequalities within and among countries; SDG 11: Make cities and human settlements inclusive, safe, resilient and sustainable)^29^ in marginalized children at risk of worse outcomes. In a context of finite resources and constrained health spending, we aim to illustrate the importance and evaluate the clinical and cost‐effectiveness of public health interventions from LMICs with close public participation to improve child health outcomes globally.

Our experience of employing a participatory, community‐based approach in the NEON Intervention Development Phase highlighted the importance and value of close collaboration with the community and partnership with health and social care practitioners. Obtaining continuous input from our target community at various points facilitated the design and refinement of our intervention by providing cultural oversight and accountability. Furthermore, it is envisaged that NEON Recipes Book and Community Assets Map tools have the potential to become trusted community resources, also in the long term. The next phase of the NEON programme will be the pilot feasibility RCT phase. This will be when the PLA intervention and toolkit will be adapted and piloted with the aim of empowering South Asian carers to introduce optimal healthy feeding practices for infants aged <2 years old in East London.

At present, the NEON programme is targeted at communities of South Asian origin in East London, a population likely to benefit from our early intervention and life‐course approach given their considerable lifetime risk of developing diabetes, cardiovascular pathologies and dental problems.^11^ The intervention package described was codeveloped and interpreted in the context of the South Asian community of London. The intervention toolkit was validated by community participants, facilitators, NEON core team and health experts (dietician, nutritionist, PLA expert and more) through deliberative meetings and workshops where recommendations and amendments were presented, discussed and applied appropriately. One possible limitation of our study would be that the participants may not be representative of the entire South Asian community. Most participants were able to speak and understand some English and had access to digital mediums (e.g., Zoom), which may likely not be the case for all South Asian communities. Further, our contributions are based on a limited number of individuals specific to the South Asian community. Therefore, a generalization and application to other contexts, while likely, can, so far, only be postulated with caution.

The Intervention Development study aimed to highlight the steps and processes involved in developing NEON‐adapted PLA intervention through triangulation of evidence, expert opinion, NEON first‐stage findings with South Asian in East London and workshops and meetings with community facilitators and community members. Results of this study provide culturally sensitive tools validated through community participation and engagement with community members, facilitators, NEON Team and health experts who are key to aiding in the improvement of infant feeding, care and dental hygiene practices amongst South Asian communities in the London Borough of Tower Hamlets, Newham and Waltham Forest.

## 5 CONCLUSION

The Intervention Development study aimed to outline the steps and processes entailed in developing the NEON‐adapted PLA intervention with evidence triangulation, expert opinion and through the involvement of NEON first‐stage findings with South Asian in East London and workshops and meetings with community facilitators and community members. Based on the findings of this paper, the Intervention Development Phase of the NEON programme demonstrates the value of a collaborative approach between researchers, community facilitators and the target population when developing public health interventions. We recommend that interventions to promote infant feeding, care and dental hygiene practices should be codeveloped with communities. Recognizing and taking into account both social and cultural norms may be of particular value for infants from ethnically diverse communities to develop interventions that are both effective in and accepted by these communities. Furthermore, as the communities are considered the codevelopers, these products will become the community's trusted resources, which will also lead to community ownership and ensure sustainability.

## AUTHOR CONTRIBUTIONS

Monica Lakhanpaul, Logan Manikam, Michelle Heys, Neha Batura, Clare Llewellyn, Andrew Hayward and Shereen Allaham conceived the original concept of the study and designed the research methodology. Shereen Allaham carried out the Intervention Development meetings, workshops and codevelopment of the intervention toolkit and wrote the first draft of the paper. Logan Manikam, Monica Lakhanpaul, Shereen Allaham, Neha Batura, Clare Llewellyn, Andrew Hayward, Rajalakshmi Lakshman, Jenny Gilmour, Kelley Webb Martin, Carol Irish, Mfon Archibong, Chanel Edwards, Corinne Clarkson, Mary Marsh, Daley Delceta and Amanda Nutkins validated the study and revised the manuscript critically for important intellectual content. Isabel‐Catherine Demel was involved in drafting materials for the NEON meetings and workshops of the Intervention Development Phase. Ummi Aisha Bello, Maryan Naman and Isabel‐Catherine Demel contributed to the manuscript writing and prepared it for submission. Logan Manikam, Shereen Allaham and Monica Lakhanpaul had primary responsibility for the final content. All authors read and contributed to reviewing the study data, the designing of the manuscript and the approval of the final manuscript.

## CONFLICT OF INTEREST

The authors declare no conflict of interest.

## ETHICS STATEMENT

This study has obtained ethical approval from UCL Research Ethics Committee (Ethics ID 17269/001). Participant information sheets and consent forms have been provided to community members and those expressing interest. Community participants have agreed to participate and given audio/video consent before their participation in the workshops. Verbal consent was witnessed and formally recorded. All participants have been informed of their right to freely withdraw from the study at any time. Confidentiality of personal data has been ensured through the use of anonymisation techniques as stated in the Data Protection Act (1998) and in line with the General Data Protection Regulation (2018). All participant data has been anonymized and stored on an encrypted password‐protected computer. Data can only be accessed by authorized research personnel.

## Supporting information

Legend for supplementary file ‐ *Conceptual map illustrates the factors that influence infant feeding, care, and dental hygiene practices. Factors in red delineate what the NEON Intervention Toolkit aims to address*.

## Data Availability

The data supporting the findings of this study are available upon reasonable request from the corresponding author. It is not publicly available due to privacy and ethical restrictions.
