# Supplementary figures and images for: Developing a community facilitator‐led participatory learning and action women's group intervention to improve infant feeding, care and dental hygiene practices in South Asian infants: NEON programme
Source: Health Expect. 2022 Jul 27;25(5):2416–30. doi: 10.1111/hex.13557 (PMC9615055; doi:10.1111/hex.13557)

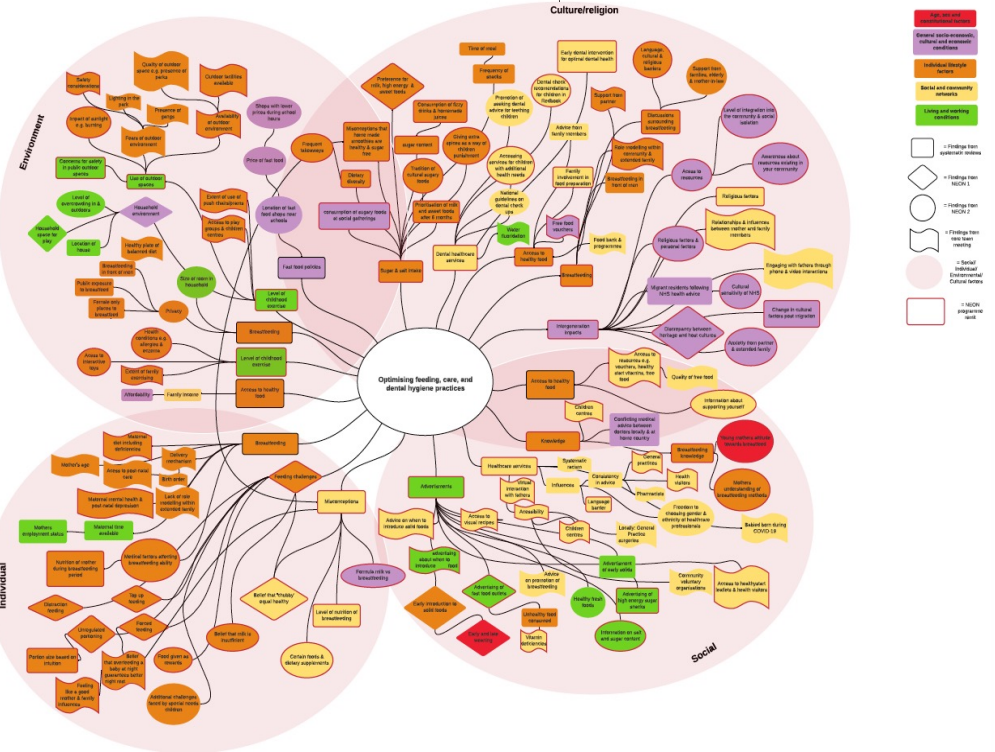

Supplement: Supplementary file 1 — Legend for supplementary file ‐ Conceptual map illustrates the factors that influence infant feeding, care, and dental hygiene practices. Factors in red delineate what the NEON Intervention Toolkit aims to address. [file HEX-25--s001.pdf]
